# Functional Connectivity in the Brain Estimated by Analysis of Gamma Events

**DOI:** 10.1371/journal.pone.0085900

**Published:** 2014-01-21

**Authors:** Anatol Bragin, Joel Almajano, Farshad Kheiri, Jerome Engel

**Affiliations:** 1 Department of Neurology, David Geffen School of Medicine at UCLA, Los Angeles, California, United States of America; 2 Department of Neurobiology, David Geffen School of Medicine at UCLA, Los Angeles, California, United States of America; 3 Department of Psychiatry and Biobehavioral Scienses, David Geffen School of Medicine at UCLA, Los Angeles, California, United States of America; 4 Brain Research Institute, David Geffen School of Medicine at UCLA, Los Angeles, California, United States of America; Universiteit Gent, Belgium

## Abstract

It is known that gamma activity is generated by local networks. In this paper we introduced a new approach for estimation of functional connectivity between neuronal networks by measuring temporal relations between peaks of gamma event amplitudes. We have shown in freely moving rats that gamma events recorded between electrodes 1.5 mm apart in the majority of cases, are generated by different neuronal modules interfering with each other. The map of functional connectivity between brain areas during the resting state, created based on gamma event temporal relationships is in agreement with anatomical connections and with maps described by fMRI methods during the resting state. The transition from the resting state to exploratory activity is accompanied by decreased functional connectivity between most brain areas. Our data suggest that functional connectivity between interhemispheric areas depends on GABAergic transmission, while intrahemispheric functional connectivity is kainate receptor dependent. This approach presents opportunities for merging electrographic and fMRI data on brain functional connectivity in normal and pathological conditions.

## Introduction

Cognitive processes in the brain require coordinated activity of distributed neuronal networks in functionally specialized brain areas [1]. In each brain area, different types of neurons are organized in functional modules with varying density of local and long connections [2], [3], [4]. Brain electrical activity is represented by a variety of oscillatory activities in frequency bands from 0.01 Hz to 1000 Hz [5], and oscillations in a specific frequency bands have different mechanisms of generation [6], [7], [8], [9]. Gamma activity is observed in many brain areas during different behavioral states and there are several hypotheses about its functional role [6], [8]. The most popular approach to analysis of gamma activity is to concentrate on the oscillatory aspect of the process.

We have used an approach that views gamma activity as a sequence of events, to measure functional connectivity among neighboring and remote modules of neurons. There are 2 reasons that we have chosen this frequency band in our experiments: 1) Gamma waves have relatively low amplitude and contribute little to volume conductivity [7], [9]. 2) There are several publications indicating that gamma events are generated by local networks [7], [9], [10], [11] see for review [2], [6], [12], [13], [14]. The duration and amplitude of gamma waves are modulated by stimulus and behavioral state and vary rapidly from one cycle to the next [15].

We have asked the following questions in this study: How big are modules generating gamma events? Does the temporal correlation of gamma events among modules reflect functional connectivity? If so, what are the properties of these functional connections and how are they related to the functional connectivity pattern described in functional magnetic resonance imaging (fMRI) studies?

## Methods

All procedures described in this study were approved by the University of California at Los Angeles Institutional Animal Care and Use Committee. Experiments were performed on 28 adult male (200–250 g) Sprague-Dawley rats.

### Microelectrode Implantation

During microelectrode implantation rats were anesthetized with 5% isoflurane. A pair of tungsten micro-wires (50 µm OD) with 1.5 mm distance between the tips were implanted bilaterally into the following brain areas: 1) Left (LAD) and right (RAD) dentate gyrus of the anterior hippocampal complex (coordinates (AP = −3.5, ML = 2.0, DV = 4.5), 2) Left (LPD) and right (RPD) dentate gyrus of the posterior hippocampal complex (coordinates (AP = −6.0, ML = 4.5, DV = 4.0) 3) Left (LEC) and right (REC) entorhinal cortex (coordinates AP = −8.0, ML = 5.0, DV = 7.0), 4) Left (LPir) and right (RPir) piriform cortex (coordinates (AP = 1.0; ML = 4.5; DV = 7.5). In 11 of 28 rats the LPir recording site was replaced by implantation of a cannula for microinjection of drugs and a recording microelectrode in the right posterior CA3 area of hippocampus (coordinates AP = −5.6, ML = 4.0 DV = 5.0). The reference and ground electrodes were placed in the cerebellum near the mid line at a distance 1 mm from each other. This close proximity of the reference electrode to the ground electrode did not not generate visible local field activity [16].

### Baseline Data Acquisition

Experiments were carried out under freely moving conditions. Beginning one week after surgery rats were plugged in to MOSFET input operational amplifiers mounted to cable connectors to record baseline activity for 8 hours. Brain electrical activity was recorded with the frequency-band 0.1 Hz to 3.0 kHz and sampled at 10.0 kHz/channel with 12 bit precision on a Pentium PC using Run Technologies (Mission Viejo, CA) DataPac software simultaneously with video recording. On the next day animals were plugged in again and after 2 hours of baseline recordings different drugs were injected into the right hippocampus (see details below).

### Drug Application

We have analyzed the effect of two drugs on the pattern of functional connectivity (FC): bicuculline (BIC), and kainic acid (KA), in the following sequence: baseline activity->3 days break -> bicuculline ->3 days break -> KA. After KA injection experiments were terminated because of the long term effect of KA.

BIC and KA were injected into the CA3 area of hippocampus through an implanted cannula (see details above) with a 10 µl Hamilton syringe with flow rate of 0.1 µl/10 s in a dosage 100 µM/2 µl for BIC and 9.3 µM/0.4 µl for KA. All recordings were performed 0.5–3.0 hours after injection.

### Morphological Controls

After completion of electrophysiological experiments animals were anesthetized with an overdose of Nembutal and 5 minutes after the heart stopped beating animals were plugged in to the amplifier in order to perform post-mortem recordings for 3–5 minutes to obtain of the noise activity of the electrical system. After this animals were perfused with 4% of paraformaldehyde and the brains were sliced in 60 um sections to identify the location of recording electrodes.

### Selection of Files for Analysis

From a total 28 rats, subgroups with no less than 11 animals were formed for different experiments. To obtain data under comparable conditions, we selected three files in of 3 minute duration on each animal from two behavioral states: 1) Resting-sitting state periods when rat was immobile eyes close, sitting quietly with absence of regular theta waves in the electrical activity and 2) during exploration activity, which is characterized by the existence of theta activity (TA). These states were verified by video recording and identified on the basis of amplitude of EEG and dominance of slow waves (<1 Hz) for the Resting state and theta activity for the TA state, on the power spectrograms. After BIC, KA injection, 3 minute files were selected before the 1^st^ seizure occurrence.

### Data Analysis

Functional connectivity during gamma activity was estimated based on the temporal relation between local maxima of gamma events recorded from any two recording sites. A detailed description of this approach is provided in an earlier publication [17].

#### Selection of gamma events

As a first step the data were down sampled up to 1.0 kHz and filtered. The data recorded from dead subjects were considered to be the system noise. This noise was subtracted from the signal using a Wiener filter, which changed the tuning number (signal processing Matlab toolbox). The ratio the noise to the original signal was estimated based on the ratio of the amplitude of the noise to the recorded signal, and the window size was selected large enough to contain four periods of the lowest frequency of gamma events (30 ms). After noise cancelation a FIR bandpass filter (30–55 Hz) was applied to extract the gamma frequency and then the local maxima of gamma event amplitudes were detected using the detection algorithm in the Matlab toolbox. To detect local maxima of gamma events a function in Maltab named “findpeaks” was used. This function uses the simple algorithm based on the first and second derivations of the signal, It also allows choice of a minimum interval between two consecutive peaks included in the analysis. The detected peaks were called “gamma events”. Inter-peak intervals of 18–30 ms were equivalent to the gamma activity.

#### Calculation of functional connectivity

To evaluate the relationship among detected events, we used peri-event histograms which visualized the rate and timing of events in relation to each other. For each event in the reference channel the number of events of the target channel within a ±30 msec window was calculated. The procedure was repeated for all N detected events in the reference channel and a time histogram was created. Details of the algorithm can be found [18], [19]. Based on the Nyquist rate, the highest detectable frequency of events should be half the sampling rate (1000 Hz/2 = 500 Hz). Two events can be separated from each other by a time resolution of 2 ms (1/500 Hz). Therefore, we chose 2 ms as the bin size. The time window (L) during which a related event could occur was chosen as 1/fmin (fmin is the minimum frequency in each frequency range). For the selected frequency band, fmin is 30 Hz, thus L is 1/30 Hz≈34 ms. According to a rule of thumb used by statisticians, to produce a valid histogram we need at least 30 data points for each bin interval. Therefore, 1020( = 34 bins×30 events) events need to be collected with minimum file duration of 24 seconds (≈1020 events/42.5 Hz).

The two channels from the histogram were considered functionally connected when there was a significant peak in the peri-event histogram. The strength of the peak in the histogram was measured using Shannon entropy (S). For the histogram with *N* bins, the *p_i_* was the probability of an event belonging to the *i^th^* bin. *S* is defined as:
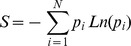



The lower the *S*, the more certainty in the data (stronger connectivity between the two channels involved in the histogram). Maximum *S* indicates uniform distribution. With uniform distribution, all events have the same likelihood of occurrence (*p_i_* = *1/N* ). Therefore *S_max_* is:
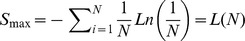



We defined a “*connectivity index”* as the strength of connectivity between two recording channels, which varies from ‘0’ to ‘1’ (‘1’ means fully connected and ‘0’ means fully disconnected). A “connectivity index” (*h_ij_*) between two recorded channels (i and j) was calculated by subtracting the Shannon entropy calculated for the peri-event histogram between those channels (*s_ij_*), from its maximum (*S_max_*) divided by (S_max_):
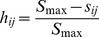



All the connectivity indexes were combined into a matrix, the “*connectivity matrix”.* The components of the connectivity matrix reflect the connectivity indices between different recording sites, i.e. the *i^th^* row *j^th^* column component shows the connectivity index between channels i and j. The connectivity matrix must be symmetrical since the connectivity index between channels i and j is equal to the connectivity factor between channels j and i. We built an MxM “connectivity matrix “for M recording channels. The component of the *i^th^* row and the *j^th^* column was *h_ij_,* which reflected the strength of connectivity between two recording channels (i and j).

To compute the resemblance between two matrices with the same rank, we used the Mantel test [20]. In the Mantel test method, to compare matrices A and B, we need to calculate the sum of the inner products of the components in each matrix (called z_m_), excluding the diagonal values:
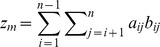
where *a_ij_* and *b_ij_* are the *i^th^* row and *j^th^* column components of the two matrices A and B respectively. However, *z_m_* can be normalized and more easily interpreted as the numbers between −1 and 1 [21]. Normalized *z_m_* is called *r_M_,*



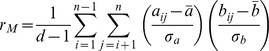
where *d* is the number of components in the upper triangular part of each matrix, *σ_a_* and *σ_b_* are the standard deviations and 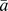 and 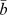 are the mean of a_ij_’s and b_ij_’s. Later on, by applying Monte Carlo randomization techniques, the p-value is estimated by permutation tests (Manly, 2007). The Mantel test gives two values, first the p-value which indicates the significance of the calculated similarity factor, and second the similarity factor. If the similarity factor is not significant, it means the two matrices are different from each other. Two matrices are considered similar, not only if their similarity value is significant but also if the value is higher than 95% of the cases produced by randomization. This threshold can be determined heuristically based on the analyzed data and it varied from one animal to another.

Randomization was performed through bootstrapping [22]. To measure the significance for the Mantel test, a normal approximation to determine significance is the permutations of all elements (for n elements, n!). 1000 randomizations have been proposed as a minimum for estimating a significance level of about 0.05 and 5000 for the significance level of 0.01. For *m* files recorded during TA and *n* files during Resting state, we formed *m* and *n* connectivity matrices. These two groups of matrices were compared by the bootstrapping method.

For calculation of connectivity graphs, several periods of the EEG signal were selected from different animals and the connectivity matrix for each one was calculated and using bootstrapping techniques their significance was examined. All these calculations were performed by Matlab Statistical and Signal Processing toolboxes.

The clustering coefficient was calculated for each graph. The clustering coefficient is a ratio *N*/*M*, where *N* is the number of edges between the neighbors of *n*, and *M* is the maximum number of edges that could possibly exist between the neighbors of *n*.

#### Synchronicity index

To investigate the size of the tissue generating gamma events the event maxima were recorded with two microelectrodes separated by 1.5 mm and their number within a time window ±2 ms was calculated. To normalize recordings between different pairs of microelectrodes we introduced a *synchronicity index*, which is the ratio between numbers of events in the window ±2 ms to total number of events.

#### Gamma events and multiunit activity

For analysis of correlation between gamma events and multiunit activity the gamma events in the selected pairs were extracted as described above. The multiunit activity was extracted from high pass filtering (300 Hz) of the raw signal with a threshold of 2 standard deviations (SD) from the middle of the record. The perievent histograms were created by setting the maximum of the gamma event at the zero point.

#### Statistical analysis

Where appropriate a statistical analysis was performed using Prizm 4 (GraphPad, San Diego, CA). For samples that passed normality test, paired and unpaired t-tests, as well as one-way analysis of variance (ANOVA) followed by post hoc Bonferroni test was used where appropriate. For samples that failed the normality test, Mann–Whitney or Wilcoxon tests was applied. A p<0.05 was accepted as a level of statistically significant differences.

## Results

### Gamma Event Connectivity during the Resting State

#### Spatial dimensions of modules generating gamma events

**Figure 1:**
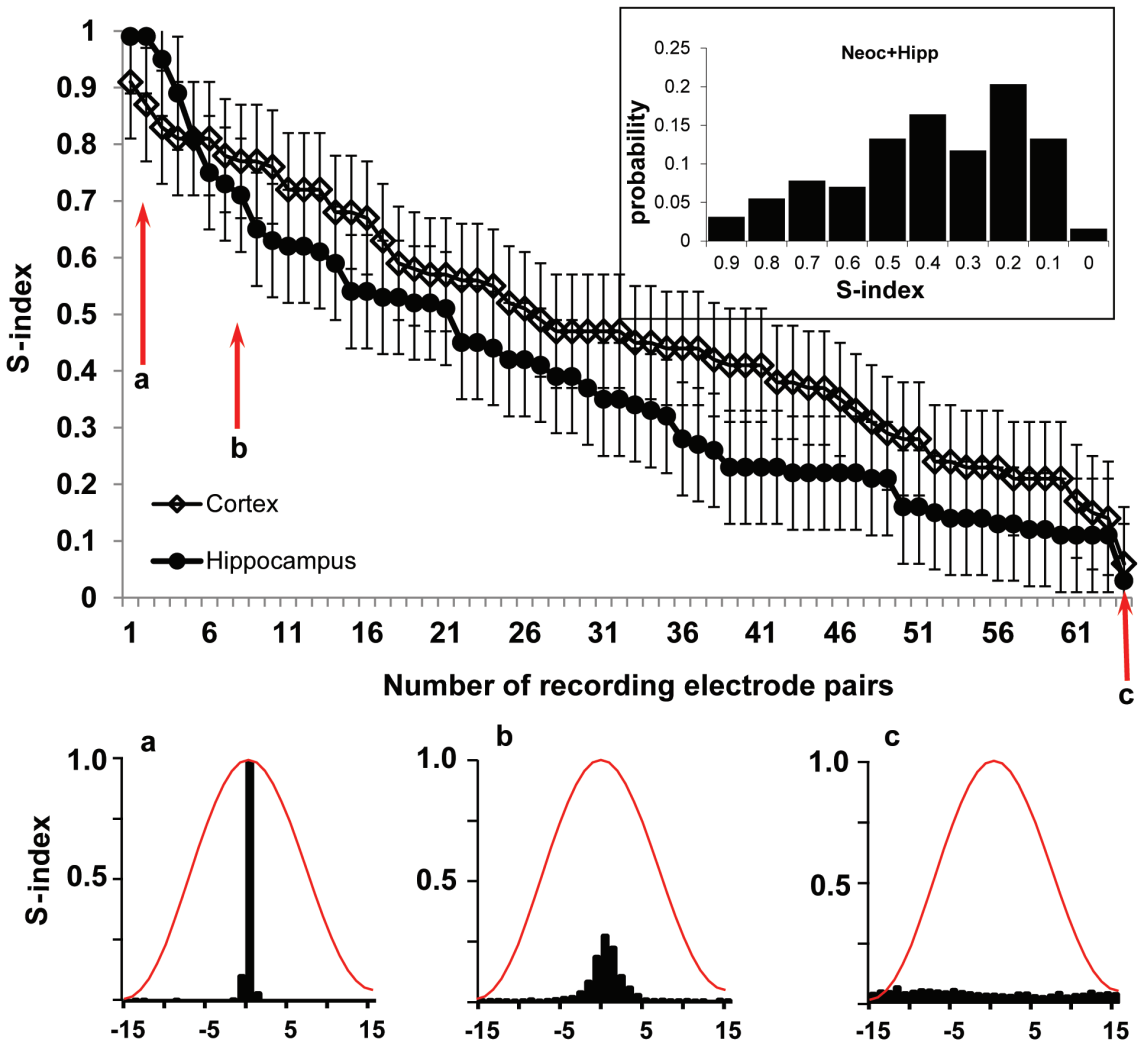
Distribution of the synchronicity Index (S-index) ranked from high to low values for zero time lag gamma in the neocortex (open diamonds) and hippocampus (filled circles) for 16 animals (n = 128 pairs). The recording sites were separated 1.5– a probability of distribution of the S-indexes. The blue line indicates the median of the data (0.42) and the dashed box outlines the area between 5^th^ and 95^th^ percentiles. **a**, **b**, and **c** – examples of perievent histograms for the S-index equal correspondingly 1.0; 0.73 and 0.05. The red line is the normalized shape of a gamma event.

Correct electrode localization was observed in all rats used for the analysis. We used a pair of 50 µm diameter microelectrodes with 1.5 mm distance between the tips as a probe for evaluation of the size of modules generating gamma events. We assumed that, if the size of the module generating gamma events is significantly larger that the distance between the tips of the microelectrodes they will record the same event and all events in the histogram between the 2 recording sites will be near the zero point. If the size of the network generating gamma events is smaller than the distance between recording sites and they are not functionally connected the histogram will not have any significant peaks. Recordings from 128 pairs recorded in 16 rats were analyzed. The results are presented in the Figure 1, where all synchronicity indices were ranked from the highest to the lowest values. The distribution of synchronicity indices was similar for pairs of microelectrodes implanted into neocortex and hippocampus. The graph in the inset to Figure 1 illustrates that the lowest (0.01–0.03) probability of occurrence of synchronicity indices is for the lowest (0.01–0,1) and highest (0.9–1.0) values, which correspondingly are within the lowest and highest 5^th^ percentile of the data. The synchronicity indices with values close to “1″ and “0″ were observed in only 3%. In 97% of cases the synchronicity indices varied between 0.2 to 0.9. On the basis of these data we suggest that the size of modules generating gamma events is comparable or smaller that the distant between recording sites and there is a very small chance that microelectrodes with 1.5 mm separation between the tips could be located within the same module.

#### Functional connectivity between distant brain areas

FC were analyzed between 308 pairs of brain areas recorded in 11 animals and 9 brain areas selected for analysis of functional connections. Bilateral placements were: left and right piriform cortices, left and right anterior and posterior dentate gyri, left and right entorhinal cortices. In addition to these areas, electrodes were implanted in the CA3 area of the right hippocampus where local injection of bicuculline and kainic acid was performed (see below). The question was asked how activation of the inhibitory or excitatory synaptic transmission effects the functional connections of the injected area.

**Figure 2:**
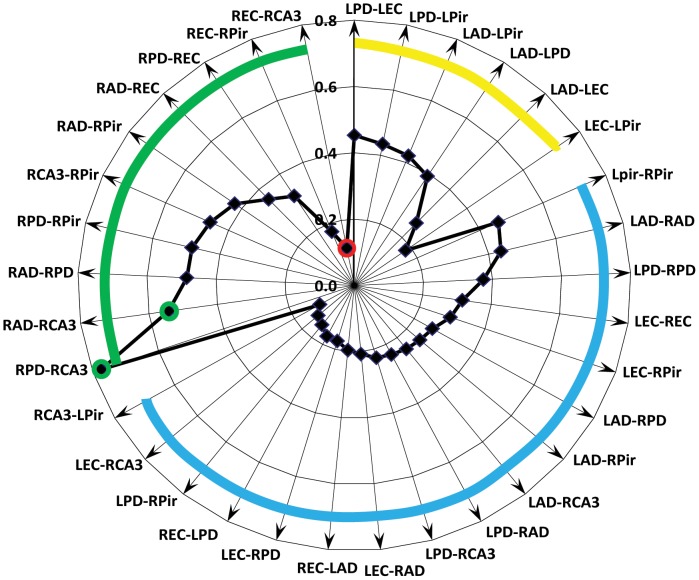
Radar graph illustrating the average connectivity index between different brain areas during the Resting state (n = 11). The green circles indicates the highest (>0.5) values of the connectivity index, which is between RAD-CA3 and RPD-CA3. They correspond to morphological data of high connectivity within the hippocampal circuitry. The red circle indicates the lowest (<0.2) value of the connectivity index, which is between the right CA3 area and ipsilateral entorhinal cortex. The green thick line outlines the connectivity index between recording pairs in the right hemisphere, yellow in the left, and blue in the interhemispheric recording pairs.

The pattern of the strength of FC between brain areas was consistent in several measurements in the same animal, but it was different from animal to animal. In average, our experiments showed a correspondence of gamma event FC with known morphological connections (Figure 2). The highest connectivity indices were observed between area CA3 of hippocampus and ipsilateral dentate gyri (green circles), which have strong morphological connections with each other. The lowest FC were observed between brain areas that do not have direct morphological connections: piriform and entorhinal cortices and entorhinal cortices and contralateral dentate gyri. Surprisingly low FC were observed between entorhinal cortex and ipsilateral CA3 area of hippocampus (red circle), although there are direct connections to the distal dendrites of CA3 from entorhinal cortex. In the group of inter-hemispheric recording sites the highest connectivity index was observed between those that were located in homotopically (piriform cortices, dentate gyri and entorhinal cortices) and lowest between brain areas that do not have direct anatomical connections.

#### Gamma event related functional connectivity and neuronal discharges

**Figure 3:**
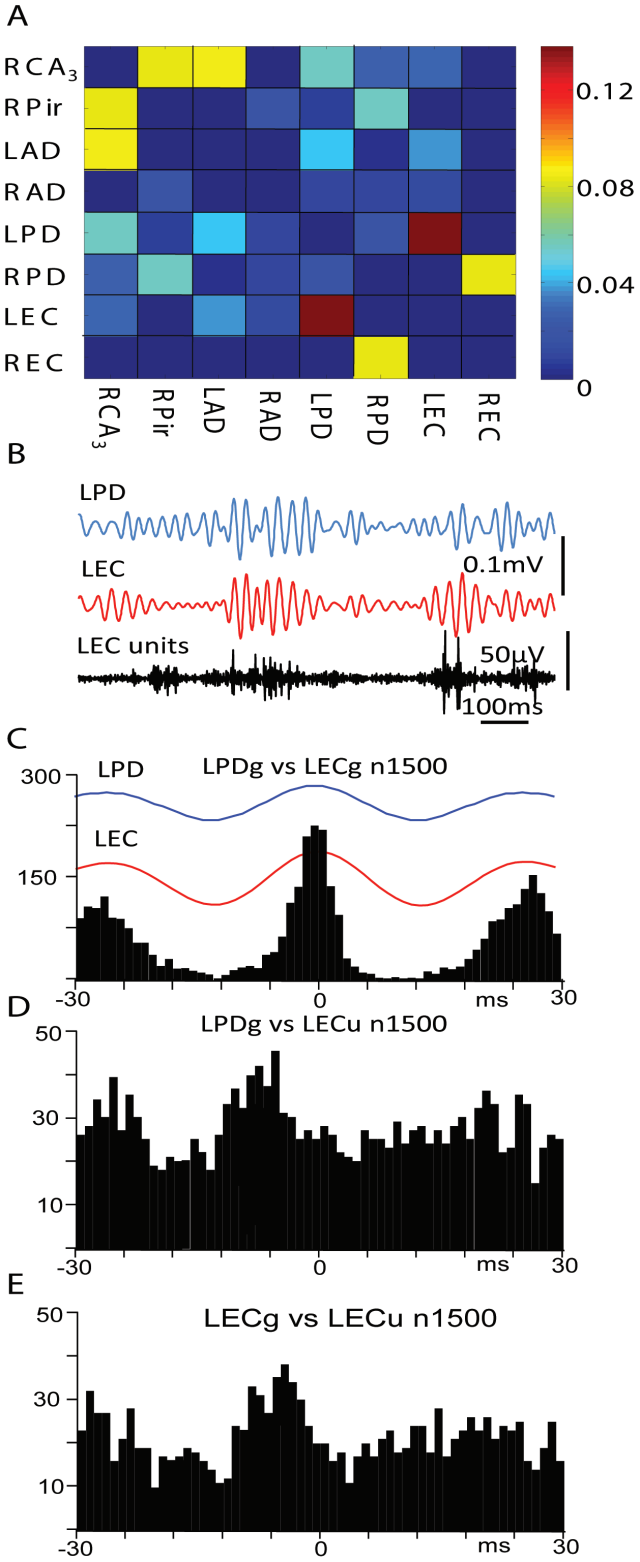
Correlation of multiunit discharges in the entorhinal cortex with local and remote gamma events during the RS. A. Matrix of rat #159 showing that gamma events in the left posterior dentate gyrus (LPD) and in the left entorhinal cortex (LEC) have the highest connectivity index. Calibration bar for the connectivity matrix values is presented on the right. B. Examples of gamma activity in the LPD and LEC and multiunit activity in the LEC. C. Perievent histogram of gamma events recorded in the LEC versus 1500 of the LPD gamma wavelets. The average of these events is shown at the top. D and E. Perievent histograms of multiunit activity recorded in the LEC versus gamma events correspondingly in the LPD and LEC.

From total 812 pairs recorded in 28 animals’ during the resting state 51 pairs with the highest and 51 with the lowest connectivity index were selected for analysis of relationships between multiunit discharges in one area and gamma events recorded in another brain area. In the group with lowest connectivity index all multiunit recordings showed increased discharges with gamma events recorded with the same microelectrode, but none of the selected pairs showed a significant relationship between multiunit activity and gamma events recorded in remote areas. In the group with the highest connectivity index in 12 pairs (23.5%) significantly increased multiunit discharges during both local and distant gamma events was observed. This increase occurred either at the negative peak or on the ascending part of the gamma events. An example of such an increase is shown in the Figure 3. According to the matrix presented in part A, the highest connectivity index (red squares) in this rat was between left posterior dentate gyrus (LPD) and left entorhinal cortex (LEC). Averaged gamma events in these areas occurred with a zero time lag and a range of ±5 msec based on perievent histogram (Figure 3 B,C). The frequency of multiunit discharges in LPD increased during local gamma events, but did not change significantly during LEC gamma events (not shown). Multiunit discharges in the LEC increased their frequency of discharges on the ascending part of both LPD and LEC gamma events (Figure 3 D,E).

### Changes in FC between Resting and Theta States

In these experiments we asked the following questions: a) does the pattern of FC change from one behavioral state to another? b) if yes, is there any consistency (rules) in change of FC between specific brain areas when the brain “shifts” from the Resting state to the Theta state?

**Figure 4:**
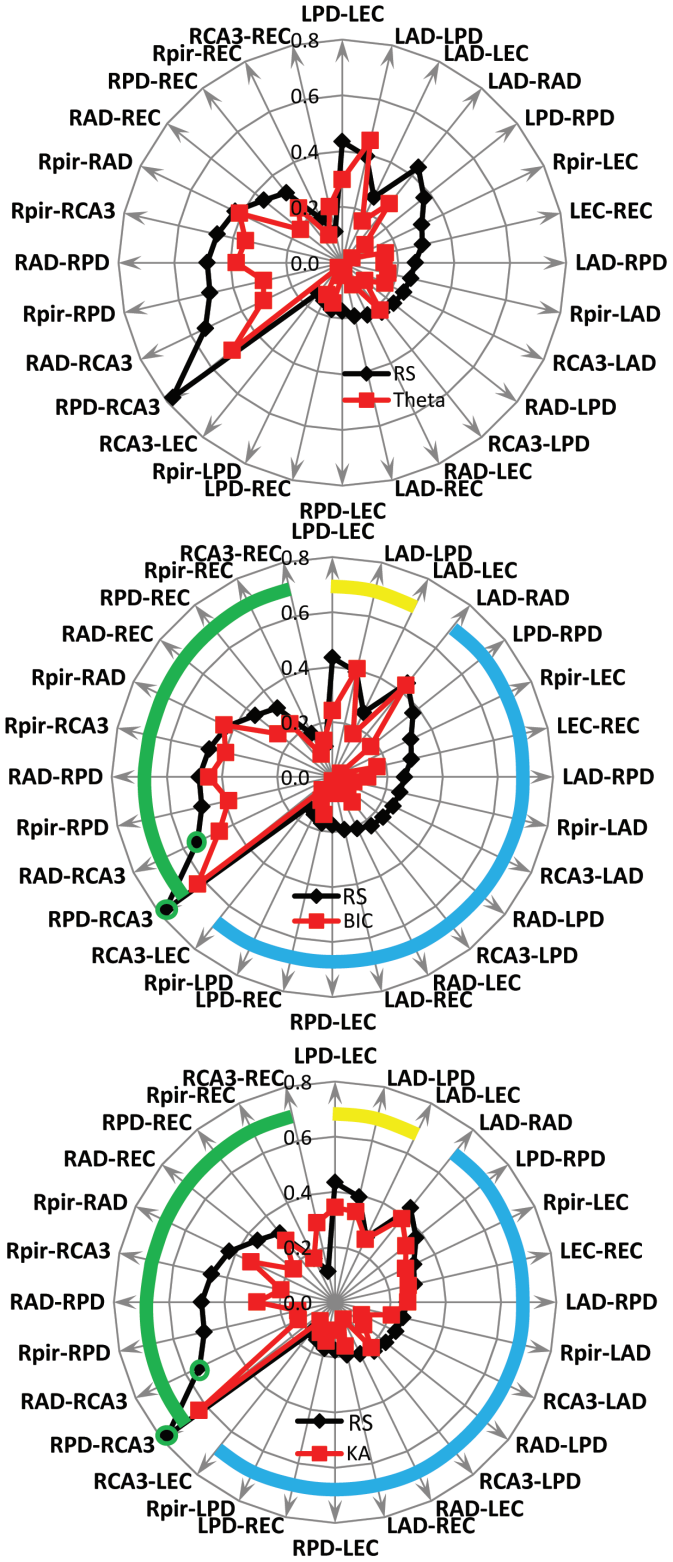
Radar graphs illustrating the average connectivity index (n = 11) between different brain areas during the Resting state (black labels) and other states of the brain (red labels). A. – during the Theta state; B– after intrahippocampal bicuculline (BIC) and and C - after intrahippocampal kainic acid (KA) injection. The green line outlines the connectivity index between recording sites in the right hemisphere, yellow in the left, and blue interhemispheric recording pairs. Statistically significant changes in the functional connectivity index p<0.05 (t-test) are indicated by asterisks.

In this series of experiments FC were analyzed in the same group of 11 animals as during the Resting state. Compared to the Resting state, the pattern of FC during the theta state changed in all rats and pattern of changes was specific for each animal. In majority cases there was a decrease of FC between both ipsilateral and bilateral recording sites (Figure 4, A).

In order to identify the synaptic networks that participate in the decrease of functional connectivity during the Theta state we compared functional connectivity during the Resting state and after blockage of GABAA and kainite receptors. Intrahippocampal injection of bicuculline lead to decreased FC between bilateral recoding sites (Figure 4, B) while intrahippocampal injection of kainic acid lead to decrease of FC between ipsilateral recording sites (Figure 4 C).

The cluster coefficient of FC as in the theta state and after application of different pharmacological agents did not change significantly compared to that observed during Resting state (data not shown). One explanation for this could be that the cluster coefficient is not sensitive enough to distinguish the difference between different states with a relatively small number of recording sites.

## Discussion

In our previous publication [17] we introduced the approach for analysis of gamma events and demonstrated that the pattern of functional connections calculated by the analysis of temporal relations of gamma events is animal specific and is different for SWS and Theta states for each animal. However, within each state this pattern is stable for a period of up to 4 days. In this study we further investigated the properties of networks generating zero-time lag gamma events and estimated the size of the modules generating these events.

The data presented in this paper illustrate that a view of gamma activity as a sequence of events opens a new perspective in analysis of functional connections between local and remote brain areas. Our data on synchronicity index between pairs of microelectrodes with fixed 1.5 mm separation have confirmed earlier publications that gamma activity is generated by local modules [7], [9], [10], [11]. The detailed morphological analysis of the rat barrel field, performed in Sakmann’s Laboratory [23], found the dimension of the module to be about a 2 mm cylinder, 0.5 mm in diameter. However, similar description of morphological moduli in other brain areas is absent. Sirota and co-authors (2008), using multi-shank silicone probes found that neocortical gamma oscillations could be observed along a column or in any single layer of the neocortex. In our study a synchronicity index near “1″ was observed in a small percentage (2–3%) of microelectrode pairs, which indicate that the size of some modules generating gamma events is near the distance between microelectrodes. In the majority of cases the synchronicity index varied between 0.2–0.9 indicating that our two-microelectrode probe was recorded gamma events from two neighboring modules. These neighboring modules may have strong or weak functional connectivity with each other although the physical distance between them is the same (∼1.5 mm). Our unpublished observations show that in the current electrode montage, when the reference electrode was located near the sagittal suture of the cerebellum at a distance of one millimeter from the ground electrode (see the Methods section), the amplitude of the signal recorded from the reference electrode did not exceed the amplitude of the noise recorded during post-mortem conditions.

One of the novelties of our data is that gamma events recorded in different brain areas can occur with zero-time lag or within an event time window during the Resting state. In many previous studies zero time lag events were rejected from analysis as a source of potential artifacts. In this study zero-time lag events were considered as an indicator of functional connectivity. We have assumed that if these distant neuronal modules belong to the same broad functional network, their gamma events, which reflect the input to the module, should occur within the time window of the event. Such synchrony could also be the result of common input to these modules from a distant source not included in the recording electrode array. Taking into account the large number of neurons within a single module (∼15,000 [23]) and the large amount of synaptic connections (∼50,000,000 [23]), there is also the probability that gamma events may be coupled between these modules due to other unknown reasons of flow of electrical activity.

The occurrence of zero-time lag gamma activity driven by sensory stimulation, originally observed by Singer’s Laboratory [24], [25], [26], is hypothesized to reflect binding processes during the receipt of sensory signals. Similar “0″ time lag gamma activity may occur in the absence of sensory stimulation reflecting other mental processes for example during storage or retrieval of information [2], [14], [27].

During the Resting state the value of the connectivity index corresponds to the morphological data and functional connections that were observed in fMRI studies during the resting state. The highest connectivity index was observed between the CA3 area of hippocampus and the ipsilateral dentate gyrus, which are known to have strong morphological connections. The highest connectivity between bilateral recording sites was observed between homotopical areas: piriform cortex, dentate gyrus and entorhinal cortex. This is also in agreement with fMRI data showing high functional connectivity between homotopically located areas during the resting state [28], [29], [30], [31], [32], [33]. A surprisingly low connectivity index was observed between the entorhinal cortex and the ipsilateral CA3 area, which received direct projections from the entorhinal cortex. There are two possible explanations for this: 1) synapses from entorhinal cortex terminate on the distal dendrites of CA3 area and make only a small contribution to the PSP on the somata of pyramidal cells; or 2) CA3 is itself a generator of gamma activity, which may have during the Resting state low correlation with gamma activity generated in the entorhinal cortex. Functional connectivity estimated by the temporal relationship between gamma events was confirmed by phase lag discharges of multiunit activity observed in 23.5% of pairs with high connectivity indices, but not between recording pairs with low connectivity indices.

Our data showed that, as in the human (Chu et al., 2012), the pattern of functional connections in rats varied from animal to animal. However our method has a higher temporal sensitivity compared to the method applied in the human. To obtain a consistent picture of functional connectivity they would need to have analyzed a minimum of 100 seconds of data [34]. With our method [17], twenty four seconds of recording was enough to obtain a stable matrix of connectivity indices.

Both intra-hemispheric and inter-hemispheric functional connections decreased during the Theta state compared to the Resting state (Figure 4A). This might indicate that sensory signals that are acquired during exploratory activity form focused spatial networks that inhibit the broader networks existing during Resting state. The mechanisms of suppression of broad functional networks during transition to the Theta state are not clear and our results suggest that they are different for intra-hemispheric and inter-hemispheric functional connections. In our experiments intrahippocampal injection of bicuculline lead to suppression of inter-hemispheric but not intra-hemispheric functional connections (Figure 4B), while injection of KA suppressed intra-hemispheric but not inter-hemispheric connections (Figure 4C). These data do not confirm the initial proposal of Hubel and Wiesel [35] that inter-hemispheric fibers serve the same functions as intra-hemispheric fibers. It is not clear how activation of the same area of hippocampus by a GABAA receptor antagonist and a kainite receptor agonist can cause different effects in functional connectivity. We suggest that the suppression of inter-hemispheric functional connections by bicuculline reflects an initial effect on the feed-forward inhibition between right and left hemispheres. However, feed-forward inhibition is described between multiple brain areas ipsi- and contralateral [36], [37], [38], [39] during normal and pathological conditions. Kainic acid injected in the same area may activate a different subset of neurons that do not participate in transcallosal transfer of electrical activity. More detailed pharmacological analysis is required in order to understand the potential mechanisms underlying this differential suppression of functional connections.

In conclusion our preliminary observations indicate that viewing gamma activity as a sequence of gamma events opens a new way to compare functional connections observed in fMRI studies. One of the similarities of these two approaches is that both use activity within a local domain as a unit for analysis of functional relationships: voxels in the case of fMRI and gamma events generated by local circuits in this study. This approach may elucidate electrographic correlates of functional connectivity detected in fMRI studies. These two approaches will complement each other.
